# *GSTP1* methylation and polymorphism increase the risk of breast cancer and the effects of diet and lifestyle in breast cancer patients

**DOI:** 10.3892/etm.2012.710

**Published:** 2012-09-17

**Authors:** ANUBHA SAXENA, VARINDERPAL S. DHILLON, MOHAMMAD SHAHID, HESHAM SALEH KHALIL, MADHU RANI, TRINATH PRASAD DAS, SURESH HEDAU, ARIF HUSSAIN, RAZA ALI NAQVI, S.V.S. DEO, N.K. SHUKLA, B.C. DAS, SYED AKHTAR HUSAIN

**Affiliations:** 1 Human Genetics Laboratory, Department of Biosciences, Jamia Millia Islamia (A Central University), New Delhi, India;; 2 Unit of Experimental Medicine, Christian de Duve Institute of Cellular Pathology, Université Catholique de Louvain, Bruxelles, Belgium;; 3 CSIRO Food and Nutritional Sciences, Gate 13, Kintore Avenue, Adelaide, Australia;; 4 Dentistry Genetics Laboratory, Department of Oral and Maxillofacial Surgery and Diagnostic Science, College of Dentistry;; 5 Department of Biochemistry and Molecular Biology, College of Medicine, Salman bin Abdulaziz University/formerly Alkharj University, Alkharj;; 6 College of Dentistry, King Saud University, Riyadh, Kingdom of Saudi Arabia;; 7 School of Biotechnology, Jawaharlal Nehru University, New Delhi;; 8 Institute of Cytology and Preventive Oncology, Noida, Uttar Pradesh, India;; 9 Department of Biotechnology, Manipal University, Dubai, United Arab Emirates;; 10 Department of Surgical Oncology, Institute Rotary Cancer Hospital, All India Institute of Medical Sciences, New Delhi, India

**Keywords:** GSTP1, polymorphism, methylation, expression

## Abstract

Glutathione *S*-transferases (GSTs) are an important group of isoenzymes that play an essential role in the detoxification of carcinogens. Polymorphism at exon 5 of the GST π family decreases the catalytic activity and affects the detoxification ability of the enzyme, GSTP1. *GSTP1* promoter hypermethylation and loss of expression are frequently observed in various types of carcinoma. We hypothesized that somatic epigenetic modification in homozygous mutants increases the degree to which breast cancer risk is affected by lifestyle factors and dietary habits. The present study used tumor biopsies and blood samples from 215 breast cancer patients and 215 blood samples from healthy donors. *GSTP1* polymorphism was studied using PCR-restriction fragment length polymorphism, methylation using methylation-specific PCR and loss of expression using immunohistochemistry and western blotting. No significant increase was observed in the breast cancer risk of individuals with the mutant (Val) allele [odds ratio (OR), 1.48; 95% confidence interval (CI), 0.97–2.26 for heterozygotes; OR, 1.42; 95% CI, 0.86–2.42 homozygous mutants]. *GSTP1* promoter hypermethylation was detected in one-third of tumor biopsies (74/215) and was found to be associated with a loss of expression. Genotype and tumor methylation associations were not observed. Estrogen (ER) and progesterone (PR) receptor-positive tumors had a higher methylation frequency. *GSTP1* polymorphism was not associated with increased promoter hypermethylation. The results suggest that *GSTP1* methylation is a major event in breast carcinogenesis and may act as a tumor-specific biomarker.

## Introduction

Breast cancer is the most common type of cancer that affects females (1). The interplay between genetic and epigenetic events, as well as environmental risk factors, has significant implications in the pathogenesis of breast cancers (2). Current research is concerned with identifying new genetic, epigenetic, prognostic and predictive factors. It has been shown that genetic factors (mutations in BRCA1/2) and reproductive history account for one-third of all breast cancer cases. However, in two-thirds of breast cancer cases the etiology remains unclear. Previous epidemiological studies have indicated that certain environmental agents may play an important role in the development of breast carcinomas (3). Thus, the capacity to metabolize and detoxify exogenous toxins may correlate with an individual’s susceptibility to environment-induced breast cancer. The *GSTP1* gene is involved in a wide range of detoxification reactions which protect cells from carcinogens (3,4). GSTs provide protection against the electrophilic metabolites of carcinogens and reactive oxygen species. GSTP1 is a biotransformation enzyme expressed in normal breast epithelial cells. High levels of GSTP1 have been associated with a poor prognosis in breast cancer (3).

Silencing of tumor-suppressor genes through the hyper-methylation of their promoter regions is a frequent event in carcinogenesis (5). Hypermethylation of CpG islands in the gene promoter regions of numerous tumor suppressor and DNA repair genes has been reported to be associated with events such as chromatin condensation, replication delay and gene silencing (6,7). Identification of epigenetic changes and their correlation with clinical factors may lead to improvements in breast cancer diagnosis and treatment. The 5’ region of *GSTP1* is rich in CpG islands and its methylation causes changes in expression levels in neoplastic cells, as has been reported in a number of published studies (8–15). *GSTP1* promoter hypermethylation is also associated with a loss of GSTP1 expression (13,15). Studies have investigated the methylation status of *GSTP1* in invasive breast cancer (9,16–19) and a different study revealed *GSTP1* promoter methylation to be an early event in breast cancer (10). *GSTP1* promoter methylation has also been reported to be associated with a poor prognosis in breast cancer (16).

*GSTP1* has a polymorphic site at codon 105 in exon 5, where an adenosine to guanosine (A>G) transition results in an Ile to Val substitution (*I105V*), giving rise to the GSTP1*B allele (20). Individuals with the valine allele exhibit significantly lower enzyme activity and a reduced detoxification ability (21). There are five classes of GST enzymes (α, µ, π, σ and θ) in humans. Studies have been published concerning the potential effects of the changes to the activation and detoxification abilities of GST class π enzymes on an individual’s risk of breast cancer and have established an association between the GSTP1 Ile105Val polymorphism and breast cancer risk (22–24). Previously (25,26), we demonstrated that homozygous mutant individuals have a significantly higher risk of breast cancer. Therefore, we investigated the hypothesis that epigenetic modification in homozygous mutants with reduced enzymatic activity increases the risk of breast cancer, which is further modified by various clinicopathological parameters, lifestyle factors and dietary habits.

## Materials and methods

### Subjects and sample collection

A total of 215 breast cancer tissue samples and corresponding blood samples were obtained from patients who underwent surgery at the Institute Rotary Cancer Hospital (All India Institute of Medical Sciences, New Delhi, India) between September 2006 and May 2009. An additional 215 blood samples (10 ml) from healthy donors were also obtained. Patient and control subject characteristics were as described previously (25). The study was approved by the University and Hospital’s Human Ethics Committees. Patients and controls provided written informed consent prior to their enrollment in the study. Fresh tissue samples obtained from the surgical specimens were snap-frozen in liquid nitrogen and stored at −80°C until further use. The tissue samples were categorized on the basis of tumor advancement according to the conventional staging system described previously (27,28).

### GSTP1 polymorphism analysis

The exon 5 Ile105Val polymorphism in the *GSTP1* gene was detected using PCR-restriction fragment length polymorphism (PCR-RFLP) as described previously (25). DNA from the patient and control blood samples was isolated and amplified using *GSTP1* primers and PCR products were digested with *Bsm*AI. Digested PCR products were then run on 2% agarose gel and at least 25% of the samples were repeated to confirm the results.

### GSTP1 promoter hypermethylation analysis

Genomic DNA isolated from tissue biopsies was modified by sodium bisulfite using an EZ DNA Methylation-Gold kit according to the manufacturer’s instructions (ZYMO Research Corporation, Irvine, CA, USA). The primer sequences used in the present study are listed in Table I (19). The PCR was set-up by adding 2 μl of bisulfite modified DNA to a PCR mix containing 1X PCR buffer, dNTPs, 1 unit Platinum *Taq* DNA polymerase (Life Technologies, Inc., Rockville, MD, USA) and primers (300 ng each per reaction) to make a final volume of 25 μl. Methylation-specific PCR (MSP) was carried out using the conditions described previously (25). A negative (no DNA) and positive control (universal methylated DNA) were included for each PCR set. PCR products were analyzed by running on a non-denaturing 6% polyacrylamide gel stained with ethidium bromide and visualized under UV illumination.

### Immunohistochemical analysis

Immunohistochemical analysis was performed on tissue sections placed on poly-L-lysine-coated slides. Sections were de-paraffinized in xylene and rehydrated in decreasing gradients of alcohol, followed by the inhibition of endogenous peroxidase activity (0.3% hydrogen peroxide in methanol) for 45 min. For antigen retrieval, slides were placed in citrate buffer (pH 6.0) and boiled at maximum power for 25 min in a microwave oven. The tissue sections were washed with PBS for 10 min, incubated overnight at 4°C with primary anti-GSTP1 antibody (1:200 dilution; #SC-66000; Santa Cruz Biotechnology, Inc., Santa Cruz, CA,USA) in a moist chamber according to the manufacturer’s guidelines, counterstained with hematoxylin, dehydrated and mounted for analysis. For the negative controls, sections were treated similarly with the exception of the addition of the primary antibody. Assessment of GSTP1 expression was performed under a microscope at x400 magnification.

### Western blot analysis

The amount of GSTP1 was detected by resolving the cell lysates on 12% SDS-PAGE at a 1:50 dilution. After performing SDS-PAGE, the gel was transferred to the transfer tank buffer for 20 min, followed by the transfer of proteins onto the nitrocellulose membrane using semi-dry gel transfer apparatus (Bio-Rad, Hercules, CA, USA) at 13 V for 40 min. The nitrocellulose membrane was then placed in blocking buffer (PBS, 1.0% BSA and 0.05% Tween-20) and incubated at room temperature for 1 h with constant agitation. The membrane was washed three times for 10 min per wash using wash buffer (PBS, 0.1% BSA and 0.05% Tween-20). The primary antibody anti-GSTP1was then diluted in wash buffer (1:5,000) before being added and incubated for 1 h; the membrane was washed 3 times. Secondary anti-mouse antibody diluted in wash buffer (1:5,000) was added to the membrane and incubated for 1 h at room temperature. The membrane was washed three times and developed until bands appeared. The reaction was stopped by adding 100 mM EDTA. These western blot membranes were scanned for density measurements using the Quantity One software of the Gel Documentation 2000 System (Bio-Rad).

### Statistical analysis

The odds ratios (ORs) and 95% confidence intervals (CIs) were calculated to measure the association between the *GSTP1* genotype and the risk of breast cancer. Associations between the *GSTP1* genotype and methylation status, as well as any correlation between *GSTP1* methylation and GSTP1 expression, were examined using the Chi-square test and Fisher’s exact test (SAS Institute, Cary, NC, USA).

## Results

### Polymorphism in GSTP1 genotypes

A total of 37.7% (81/215) of breast cancer patients were homozygous for the wild-type allele, 41.4% (89/215) heterozygous (Ile/Val) and 20.9% (45/215) homozygous (Val/Val) for the mutant allele. Similarly, we found the distribution of the *GSTP1* genotype in the controls to be 47% (101/215) wild-type homozygous genotypes, 34.8% (75/215) heterozygous and 18.1% (39/215) homozygous mutant genotypes. However, no significant risk of breast cancer among individuals carrying the mutant allele was observed (Table II; OR, 1.48; 95% CI, 0.97–2.26 for heterozygotes; OR, 1.42; 95% CI, 0.86–2.42 for homozygous mutants).

### GSTP1 promoter hypermethylation

**Figure 1:**
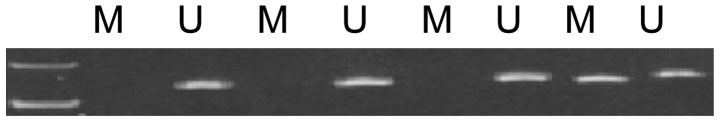
Representative results of methylation-specific PCR analysis of *GSTP1* in breast cancer patients. Amplification in M lane represents methylation, amplification in U lane represents unmethylation, and amplification in M and U lanes represents hemimethylation. M, methylated; U, unmethylated.

MSP was employed to study *GSTP1* promoter hypermethylation. Aberrant promoter hypermethylation was observed in 74 (34.4%) cases and the remaining 141 (65.6%) were unmethylated (Table III, Fig. 1). A total of 28 of the 74 methylated cases belonged to the early stage of breast cancer group while 46 were from the locally advanced disease group. Fisher’s exact test was used to determine the association between hypermethylation of the *GSTP1* gene with the clinical stages of primary breast cancer patients. In the two disease groups, *GSTP1* methylation was a significant event (in early breast cancer cases; P= 0.05; in locally advanced breast cancer; P= 0.03; Table IV). No statistically significant associations were observed between *GSTP1* methylation and various clinicopathological parameters (Table V) such as menopausal status and progesterone receptor (PR) and estrogen receptor (ER) status. The association of various lifestyle factors with *GSTP1* promoter hypermethylation was also studied, including age, smoking status, age of menarche onset, contraceptive use, family history of cancer, number of children, ER and PR status and alcohol intake. Methylation of the *GSTP1* gene was significantly associated with ER^+^/PR^+^ status (Table VI). The disease risk was increased by >2.5 times compared with ER^−^/PR^−^ tissue (OR, 2.63; 95% CI, 1.18–5.87; P=0.01; Table VI). When the results were analyzed with regard to ER and PR status with age (<50 or >50 years), the disease risk was increased by 2.5 times in the <50 years of age group (OR, 2.47; 95% CI, 0.99–6.19; Table VII). Similarly, when ER/PR status was considered in females <50 years old, the risk was increased by nearly three times when both ER and PR status were positive (OR, 2.92; 95% CI, 1.03–8.31; Table VII). However, none of the other factors were significantly associated with *GSTP1* promoter hypermethylation (data not shown).

In order to test the hypothesis that somatic epigenetic modification in homozygous mutants, combined with reduced enzymatic activity, increases the risk of breast cancer an attempt was made to determine the role of the *GSTP1* polymorphism in promoter hypermethylation. However, no significant associations of any of the three genotypes with promoter hypermethylation were observed (Table III). Therefore, it was concluded that promoter hypermethylation in homozygous mutants with reduced enzymatic activity due to the substitution mutation did not elevate the the risk of breast cancer.

### Immunohistochemical and western blot analysis of GSTP1

**Figure 2:**
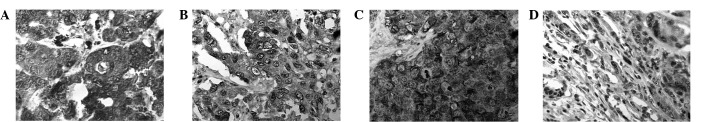
GSTP1 expression in breast cancer tissue. Section from breast carcinoma stained for GSTP1 by immunohistochemistry showing malignant cells with regions of expression and non-expression in the same tissue for GSTP1. (A) GSTP1 levels high, positive cancer cells, (B) GSTP1 levels low, positive cancer cells, (C) GSTP1-positive cancer cells and (D) GSTP1-negative cancer cells.

**Figure 3:**
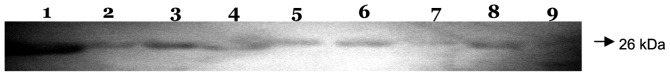
GSTP1 western blot from breast tissue biopsies. Lanes 1 and 2 show GSTP1 expression in control sample, lanes 3–9 show varying levels of GSTP1 expression in different breast carcinoma individuals.

Immunohistochemistry and western blot analysis were performed on all the tissue samples to determine the correlation between *GSTP1* hypermethylation and GSTP1 expression. Immunohistochemistry was employed to localize the protein and western blotting to quantify protein expression. Representative results of the GSTP1 immunohistochemistry and western blot analysis are shown in Figs. 2 and 3, respectively, and the results obtained in these two assays complemented each other. The immunohistochemistry results did not contradict those of the western blotting in any cases. A total of 67 tumors lacking GSTP1 expression exhibited *GSTP1* promoter hypermethylation (100%) and 7 tumors showed methylation but expressed GSTP1 (Table VIII; P<0.0005). The results indicate that methylation does not always result in a loss of GSTP1 activity and there may be other mechanisms that contribute to its activity. GSTP1 was expressed in all unmethylated samples. Strong staining and high expression levels were found in all samples of non-tumorous breast tissues.

## Discussion

Glutathione *S*-transferases are phase II metabolizing enzymes involved in the biotransformation of exogenous substances, including mutagens, carcinogens and other poisonous chemicals, and play a crucial role in the detoxification process, thereby protecting cells from these compounds (29). *GSTP1* codes for a GST π family enzyme and is located on human chromosome 11q13. Polymorphism in exon 5 (A105G) results in amino acid sequence variation which affects enzyme activity as well as its substrate specificity. Therefore, a detailed investigation of the effect of this polymorphism on an individual’s susceptibility to various environmentally generated tumors is required (30). Since a variety of environmental factors may play a decisive role in causing breast cancer in the majority of cases, the role of this polymorphism in breast cancer appears to be important. In the present study, no evidence of an increased risk of breast cancer in females carrying the variant allele (Val) at codon 105 of *GSTP1* was observed. Previously, we demonstrated that the *GSTP1* polymorphism is associated with an increased risk of breast cancer (25) and therefore, the current hypothesis was tested. Tissue samples were not available from all breast cancer cases so the present study was carried out using 215 of the 413 cases reported previously and accordingly matched controls. It is likely that the polymorphism results obtained in the present study may not represent the real scenario since the patients’ menopausal status may have affected the present results compared with the overall results obtained previously (25). In the present investigation, tissue samples were obtained from 132 pre-menopausal patients and 83 post-menopausal patients. *GSTP1* variants encode the change at position 105 from isoleucine to valine. It is predicted that this change may limit free access of the substrate to the H-site of the enzyme which is required for conjugation with glutathione, as well as thermostability (31,32). This would result in reduced activity of the polymorphic protein. However, the exact underlying biological mechanism is not completely understood (3).

Since gene methylation generally mimics the mutation pattern (33), the question of whether methylation plays a role in genetic susceptibility to breast cancer similar to that of the mutation (34) was also examined in the present study. A previous study revealed an association between *GSTP1* methylation and tumor invasion, size, sentinel lymph node metastasis and progression in breast cancer (19). In the present study, it was also demonstrated that hypermethylation of *GSTP1* occurs more frequently in advanced stage cancer cases than in early disease status cases. These findings suggest that in addition to a role in tumorigenesis, *GSTP1* methylation may also be associated with disease progression.

*GSTP1* is considered to act as a tumor-suppressor gene, leading to tumor growth when it is inactivated. It has been reported that *GSTP1* interferes with *N*-terminal c-Jun kinase signaling (35). If inactivated, *GSTP1* may act as a caretaker gene leading to additional somatic genome alterations that promote tumor growth (36). GSTP1 binds non-covalently to steroids, allowing it to act as an intracellular buffer to minimize short-term fluctuations in steroid levels. The breast is an important organ of the body which is continuously exposed to these steroids and it is therefore highly likely that estrogens act as endogenous tumor initiators in the breast tissue when *GSTP1* is inactivated by promoter methylation. *GSTP1* promoter region inactivation by hypermethylation is a common event in cancer and this epigenetic modification is often linked with a loss of GSTP1 expression (9,12,18,37,38). We observed that *GSTP1* promoter hypermethylation is present in one-third (34.4%) of human breast carcinomas, which was well within the previous reported frequency range (13 to 38.9%) (9,10,16–19). Statistical analysis revealed an inverse correlation between *GSTP1* methylation and GSTP1 expression. Methylated samples exhibited a loss of gene expression, suggesting that the silencing of the *GSTP1* gene by CpG island DNA methylation plays an important role in the development of breast cancer. In unmethylated samples an intense expression signal was obtained for all samples using expression analysis techniques (see Materials and methods). In previous studies, *GSTP1* promoter hypermethylation has been linked with gene silencing and reduced GSTP1 expression (11,38) in breast cancer cells, whereas normal mammary epithelial cells have always been found to express GSTP1 (9). In certain cases, the presence of GSTP1 expression despite *GSTP1* methylation may be attributed to a heterogeneous cell population in the tumor. Based on our findings, we propose that genotyping and methylation analysis may be considered together as a sensitive and accurate diagnostic biomarker (39).

No association was found between *GSTP1* promoter hypermethylation and the *GSTP1* genotype, although these mechanisms separately demonstrated a predisposition to breast cancer in the present study and our previous study (25). In the present study, it is notable that the methylation pattern of the *GSTP1* gene was found to be independent of age and menopausal status as reported in a previous study (40).

It has long been recognized that estrogens are linked to breast carcinogenesis (41) and that they cause genotoxic damage (42). Methylation-mediated silencing of the *GSTPl* gene may alleviate the role of estrogen as an endogenous tumor initiator. In the present study ER^+^/PR^+^ status was revealed to be significantly associated with *GSTP1* methylation in breast cancer patients from northern parts of India. Significant differences between ethnicities have been reported only in ER^−^/PR^−^ individuals aged less than 50 years old in African-American and Caucasian females (43). Methylation frequencies were also reported to be significantly higher in certain genes in Caucasian females who are more than 50 years old with ER^+^/PR^+^ than in their Korean counterparts. These differences may be explained on the basis of the ethnicity of the studied populations and possibly the associated risk factors. The present study reported similar findings, as age was significantly associated with methylation, as well as with ER and PR status, even though the sample size was small. It is known that estrogen action in normal and abnormal mammary cells is mediated by ER and that ER is overexpressed in more than two-thirds of breast cancer cases. Epigenetic modifications are potentially reversible and therefore, identification of ethnicity-specific methylation markers may be essential for cancer patient management and therapeutic intervention. Our findings suggest that ER/PR-positive tumors provide the possibility of developing biomarkers for therapeutically targeting this population (44).

In the present study, promoter hypermethylation was revealed to be the major factor underlying the loss of GSTP1 expression in breast carcinomas. However, our findings did not demonstrate that epigenetic modification in homozygous mutant individuals with reduced enzymatic activity increases the risk of breast cancer. Our previous results and those of the present study indicate that polymorphism and methylation are independent events that individually affect the risk of breast cancer. These finding may have implications for future chemotherapy regimens based on epigenetic changes and ER/PR status.

## Figures and Tables

**Table I:** Methylation-specific primer sequences for *GSTP1*.

Oligonucleotide name	Primer sequence
GSTP1 Unmethylated (Forward primer)	5′-GAT GTT TGG GGT GTA GTG GTT GTT-3′
GSTP1 Unmethylated (Reverse primer)	5′-CCA CCC CAA TAC TAA ATC ACA ACA-3′
GSTP1 Methylated (Forward primer)	5′-TTC GGG GTG TAG CGC TCG TC-3′
GSTP1 Methylated (Reverse primer)	5′-GCC CCA ATA CTA AAT CAC GAC G-3′

**Table II:** Association between the *GSTP1* genotype and breast cancer.

*GSTP1* genotype	Cases (n=215), n (%)	Controls (n=215), n (%)	OR (95% CI)	P-value^a^
Ile/Ile (A/A)	81 (37.7)	101 (47)	1 (Reference)	
Ile/Val (A/G)	89 (41.4)	75 (34.9)	1.48 (0.97–2.26)	0.07
Val/Val (G/G)	45 (20.9)	39 (18.1)	1.44 (0.86–2.42)	0.19

a Fisher’s exact test; OR, odds ratio; 95% CI, 95% confidence interval.

**Table III:** Distribution of methylated status among various genotypes of *GSTP1* in breast cancer cases.

Genotype	Methylated (n=74)	Unmethylated (n=141)	OR (95% CI)
*GSTP1* A/A (81)	29	52	1.0 (Reference)
*GSTP1* A/G (89)	30	59	0.91 (0.48–1.72)
*GSTP1* G/G (45)	15	30	0.89 (0.42–1.93)

OR, odds ratio; 95% CI, 95% confidence interval.

**Table IV:** Stage-specific methylation patterns of *GSTP1* gene.

Tumor stage	Methylated	Unmethylated	Total (%)	P-value^a^
Early	28	89	117 (54.4)	0.05
Locally advanced/metastatic	46	52	98 (45.6)	0.03
Total	74	141	215	

a Chi-square test; P-value, early/advanced cases vs. total cases.

**Table V:** *GSTP1* methylation status stratified on the basis of various clinicopathological features.

Clinicopathological features	Total	Methylated	Unmethylated	P-value^a^
Menopause status				
Premenopausal	132	50	82	0.19
Postmenopausal	83	24	59	
PR status				
Positive	121	45	76	0.39
Negative	94	29	65	
ER status				
Positive	140	54	86	0.09
Negative	75	20	55	

a Fisher’s exact test; ER, estrogen receptor; PR, progesterone receptor.

**Table VI:** Association of *GSTP1* methylation with ER and PR status

ER and PR status	Total	Methylated	Unmethylated	OR (95% CI)
ER^−^ and PR^−^	48	11	37	1 (Reference)
ER^−^ and PR^+^	38	9	29	1.04 (0.38–2.86)
ER^+^ and PR^−^	47	18	29	2.09 (0.85–5.11)
ER^+^ and PR^+^	82	36	46	2.63 (1.18–5.87)^a^

a P<0.05; OR, odds ratio; 95% CI, 95% confidence interval.

**Table VII:** *GSTP1* methylation as per ER and PR status and age distribution.

ER and PR status	Age (years)	Methylated	Unmethylated	OR (95% CI)
ER^+^ and PR^+^	>50	11	24	1 (Reference)
	≤50	25	22	2.47 (0.99–6.19)^a^
ER^+^ and PR^+^	≤50	7	18	1 (Reference)
ER^+^ and PR^+^	>50	25	22	2.92 (1.03–8.31)^a^

a P<0.05; OR, odds ratio; 95% CI, 95% confidence interval.

**Table VIII:** Expression analysis of *GSTP1* gene in breast cancer patients according to *GSTP1* methylation status.

		GSTP1 immunoexpression		
Breast carcinoma	n	Present, n (%)	Absent, n (%)	P-value
*GSTP1* methylated	74	7 (9.5)	67 (90.5)	0.0005
*GSTP1* unmethylated	141	141 (100)	0	
